# Weight-based, anti-Xa guided enoxaparin after spine trauma surgery: target attainment and safety in a retrospective cohort

**DOI:** 10.3389/fsurg.2026.1764591

**Published:** 2026-06-24

**Authors:** Dallas L. Sheinberg, David Travis Johnston, Brendan T. O'Reilly, Gabriel Galan Castro, Jawad R. Khazaal, Joseph S. Withrow, Duncan J. Trimble, Ankush Chandra, Anthony J. Lim, Derek S. Sheinberg, Jennifer Zaragoza, Wen Li, John R. Williams

**Affiliations:** 1 Department of Neurosurgery, McGovern Medical School at UTHealth Houston, Houston, TX, United States; 2 Carolina Neurosurgery & Spine Associates, Charlotte, NC, United States; 3 University of Miami Miller School of Medicine, Miami, FL, United States; 4 Department of Internal Medicine, McGovern Medical School at UTHealth Houston, Houston, TX, United States

**Keywords:** anti-factor Xa monitoring, deep vein thrombosis, enoxaparin, epidural hematoma, spinal cord injury, spinal trauma, spine surgery, venous thromboembolism

## Abstract

**Background and purpose:**

Patients undergoing surgery for traumatic spine injuries have a high risk of venous thromboembolism (VTE). Standard fixed or weight-based enoxaparin regimens may leave some patients below prophylactic anti-factor Xa (anti-Xa) target ranges. Whether anti-Xa-guided enoxaparin dose adjustment can be implemented while maintaining acceptable observed bleeding outcomes has not been studied specifically in postoperative spine trauma patients. This study evaluates anti-Xa target attainment, symptomatic VTE, and safety outcomes after anti-Xa-guided enoxaparin titration.

**Methods:**

We performed a retrospective cohort study of adults (≥18 years old) who underwent operative treatment for traumatic spine injuries at a Level I trauma center. All patients received weight-based enoxaparin starting 24 h postoperatively (30 mg every 12 h if <90 kg, 40 mg every 12 h if ≥90 kg) plus mechanical VTE prophylaxis and early mobilization. In the anti-Xa cohort, levels were obtained 4 h after the third dose (target 0.2–0.4 IU/mL) and doses were adjusted by 10 mg if outside this range. A comparison cohort received the same initial dosing without anti-Xa monitoring. The primary outcome was symptomatic postoperative VTE. Secondary outcomes included bleeding requiring transfusion, spinal epidural hematoma, hospital length of stay, and ambulation at discharge.

**Results:**

A total of 354 patients were included (170 anti-Xa; 184 fixed dose). Symptomatic VTE was rare and similar between groups (1.1% vs. 1.6%). No postoperative epidural hematomas occurred. In the anti-Xa cohort, 28% required dose escalation and 7% required more than one adjustment. In exploratory adjusted analyses, bleeding requiring transfusion and length of stay did not differ between groups.

**Conclusions:**

Weight-based enoxaparin with anti-Xa-guided titration was feasible after spine trauma surgery and identified a substantial subset of patients below the prespecified prophylactic anti-Xa target range. Protocol-based dose escalation was not associated with an observed increase in bleeding requiring transfusion, and no postoperative epidural hematomas occurred. Larger prospective studies are needed to determine whether improved anti-Xa target attainment translates into lower VTE rates.

## Introduction

Venous thromboembolism (VTE), including deep vein thrombosis (DVT) and pulmonary embolism (PE), is a major source of postoperative morbidity in spine surgery and contributes to preventable mortality, long-term disability, and increased healthcare utilization (1, 2). In large elective spine surgery cohorts that capture primarily symptomatic events, reported VTE rates are typically low, often in the range of 0.3% to 2% (3, 4). Recent studies of patients undergoing surgery for traumatic spine injuries report postoperative VTE rates of approximately 8% to 10%, even in cohorts where pharmacological prophylaxis is commonly used (5, 6). This highlights that patients undergoing surgery for traumatic spine injuries are at a much greater risk of postoperative VTE than those undergoing elective degenerative spine surgery. Mechanistically, traumatic spine injury induces endothelial disruption, systemic inflammatory activation, prolonged immobility, neurologic deficits, and polytrauma, all of which amplify thrombotic risk (7–9). Individuals with acute spinal cord injury have even higher short- and long-term VTE rates compared with other trauma populations (10, 11).

Pharmacologic prophylaxis with low molecular weight heparin (LMWH), most commonly enoxaparin, is a cornerstone of VTE prevention in this setting. However, the optimal postoperative dosing strategy in spine trauma remains uncertain. Practice patterns vary considerably because of differences in patient physiology, perceived bleeding risk, and local protocols (3, 4, 12, 13). Concern for spinal epidural hematoma, an infrequent but potentially devastating complication that can cause cord compression and irreversible neurologic decline, has contributed to surgeons' reluctance to escalate doses or individualize chemoprophylaxis regimens (2, 14–16). At the same time, several studies and contemporary trauma and spine guidelines suggest that early initiation of chemoprophylaxis, often within 24 to 48 h of injury or surgery, does not significantly increase bleeding or epidural hematoma rates and may reduce VTE events (12, 13, 17–19). These findings highlight the need to clarify how aggressively spine trauma patients can be anticoagulated without compromising neurologic safety.

A key challenge in postoperative spine trauma prophylaxis is the variable pharmacokinetic profile of enoxaparin. Multiple trauma and surgical studies have shown that fixed prophylactic regimens such as 30 mg twice daily are frequently associated with below-target anti-factor Xa (anti-Xa) levels, particularly among patients with higher body weight, altered renal function, or more severe injuries (20, 21). Weight-based dosing has been associated with higher prophylactic anti-Xa target attainment than traditional fixed-dose regimens, although a subset of high-risk patients may remain below the prophylactic target range (21, 22). Individualized approaches that use higher or weight-based dosing with anti-Xa-guided dose adjustments have demonstrated improved achievement of prophylactic target ranges in general trauma cohorts (23–28). However, these studies largely exclude postoperative spine trauma patients and were not designed around the unique balance between high thrombotic burden and high bleeding and neurologic risk that characterizes this neurosurgical population.

To our knowledge, no prior study has evaluated a weight-based enoxaparin protocol that incorporates anti-Xa-guided titration specifically in patients undergoing surgery for traumatic spine injuries. This represents a critical knowledge gap for neurosurgeons and spine trauma teams who must frequently decide whether to accept potentially subtherapeutic dosing or escalate prophylaxis despite fear of epidural hematoma. Evaluating whether individualized, anti-Xa-guided dosing can be implemented after spine trauma surgery while monitoring clinically meaningful safety outcomes has immediate implications for postoperative care pathways and institutional anticoagulation protocols.

Therefore, we conducted a retrospective cohort study comparing standard weight-based prophylaxis with a weight based, anti-Xa-guided enoxaparin titration protocol in postoperative spine trauma patients at a Level I trauma center. We examined VTE incidence, bleeding complications including epidural hematoma, and functional and hospital course outcomes. A secondary objective was to identify the frequency and predictors of below-target anti-Xa activity requiring dose escalation. Our goal is to provide spine surgeons and neurosurgeons with clinically actionable data to support individualized prophylaxis strategies in the management of traumatic spine surgery.

## Methods

### Study population and design

This retrospective cohort study was approved by the University of Texas Health Science Center at Houston Committee for the Protection of Human Subjects, with a waiver of informed consent because only de-identified data were analyzed. Adult patients (≥18 years) who underwent operative treatment for traumatic spinal injuries at a single academic Level I trauma center between July 2022 and July 2024 were screened for inclusion. Patients were excluded if injuries precluded timely venous thromboembolism (VTE) prophylaxis, an alternative anticoagulation strategy was used, they had pre-existing VTE or pre-existing therapeutic anticoagulation, they were discharged before receiving three postoperative enoxaparin doses, or they had protocol deviations that prevented cohort assignment. Eligible patients who received postoperative enoxaparin were stratified by prophylaxis pathway: a fixed-dose group that received weight-based enoxaparin without anti-Xa monitoring and an anti-Xa-guided group that underwent anti-Xa monitoring with protocol-based dose adjustment. Use of the anti-Xa-guided pathway depended on treating surgeon/service practice rather than a hospital-wide protocol change.

Clinical, demographic, and perioperative data were collected from the electronic medical record. Extracted variables included age, sex, weight, body mass index (BMI), and comorbidities such as diabetes mellitus, obesity, smoking status, history of VTE, and known clotting disorders. Injury-specific data included mechanism of injury, Glasgow Coma Scale (GCS) score on admission, presence and severity of spinal cord injury (categorized by ASIA score), and concomitant traumatic injuries, including traumatic brain injury, long bone fractures, pelvic fractures, and solid organ injuries.

Surgical variables included time from admission to operative intervention, estimated blood loss, number of vertebral levels fused, surgical approach (anterior, posterior, or circumferential), use of surgical drains, and drain output. In the anti-Xa cohort, assay values of anti-Xa were collected along with the number of enoxaparin dose adjustments required to attain the goal level between 0.2 to 0.4 IU/mL. Postoperative data collected included ambulation status prior to discharge, length of hospital stay, and occurrence of VTE, bleeding complications, or epidural hematoma.

### VTE prophylaxis protocols

All patients received mechanical prophylaxis with sequential compression devices (SCDs) beginning at admission and continuing through to discharge. Enoxaparin was administered to all patients 24 h after surgery at a weight-based dose. Patients weighing <90 kg received 30 mg subcutaneously every 12 h, while those ≥90 kg received 40 mg every 12 h.

In the anti-Xa-guided cohort, peak anti-Xa activity was measured as part of routine clinical care by the hospital clinical laboratory using the STA-Liquid Anti-Xa chromogenic assay on the STA R Max coagulation analyzer (Diagnostica Stago, Parsippany, NJ, USA). Blood samples were collected 4 h after the third consecutive postoperative enoxaparin dose in 3.2% citrated plasma tubes and processed according to institutional clinical laboratory procedures before analysis. The assay was calibrated for low-molecular-weight heparin activity and reported in IU/mL. STA-Liquid Anti-Xa has been cleared through the FDA 510(k) pathway for quantitative determination of unfractionated and low-molecular-weight heparin activity in citrated human plasma, with FDA documentation identifying the STA-Rotachrom Heparin assay as the predicate device and reporting the same chromogenic method, test principle, sample type, and STA analyzer platform (29).

The target prophylactic anti-Xa range was 0.2 to 0.4 IU/mL. If the anti-Xa level was outside the target range, the enoxaparin dose was adjusted by ±10 mg, and a repeat anti-Xa level was measured 4 h after the third dose of the modified regimen. No dose adjustments or anti-Xa monitoring were performed in the fixed-dose group.

### Outcomes

The primary outcome was symptomatic postoperative VTE, defined as deep vein thrombosis confirmed by duplex ultrasonography or pulmonary embolism confirmed by computed tomography pulmonary angiography. Routine surveillance imaging for asymptomatic VTE was not performed, and diagnostic imaging was obtained only when there was clinical concern for DVT or PE. Secondary outcomes included bleeding requiring transfusion after initiation of enoxaparin, postoperative spinal epidural hematoma (radiologically confirmed and associated with neurologic deterioration or requiring surgical evacuation), hospital length of stay, and ambulation status at discharge.

### Statistical analysis

Baseline characteristics, surgical variables, and outcomes were compared between the fixed-dose and anti-Xa groups using the Mann–Whitney U test for continuous variables and Fisher's exact test for categorical variables. Because symptomatic postoperative VTE was rare, with only five total events, regression modeling was not performed for this outcome. Regression models were limited to outcomes with sufficient event counts or continuous outcomes. Covariates for the multivariable regression models were selected *a priori* based on clinical relevance and directed acyclic graph principles, including markers of injury severity, neurologic impairment, surgical complexity, and bleeding risk. Confounders were included, whereas potential mediators and colliders were excluded. Specifically, the model for bleeding requiring transfusion adjusted for number of surgical levels, admission creatinine clearance, anticoagulant/antiplatelet use, and solid organ injury. The model for blood products transfused adjusted for number of surgical levels, anticoagulant/antiplatelet use, and creatinine clearance. The model for length of stay adjusted for Glasgow Coma Scale score, other injuries, number of surgical levels, SCI/ASIA grade, and time to operating room. The model for drain output adjusted for number of surgical levels, laminectomy defect, lumbar drain use, and anticoagulant/antiplatelet use.

Separate models were developed to identify factors associated with enoxaparin redosing and multiple re-dose requirements to achieve the prophylactic anti-Xa goal. Within the anti-Xa-guided cohort, outcomes were compared between patients who required enoxaparin dose escalation and those who did not using Fisher's exact test. Odds ratios (ORs), regression coefficients, and 95% confidence intervals (CIs) were reported where appropriate. The primary analysis was the comparison of symptomatic postoperative VTE between the anti-Xa-guided and fixed-dose cohorts. All other analyses, including secondary outcome analyses, multivariable models, predictor analyses for redosing or multiple redosing, and within-cohort dose-escalation subgroup comparisons, were considered exploratory. *P* values for exploratory analyses were interpreted descriptively and were not adjusted for multiple comparisons. All tests were two-sided. All statistical analyses were performed using R statistical software, version 4.5.1 (R Foundation for Statistical Computing, Vienna, Austria).

## Results

### Patient demographics and injury characteristics

**Figure 1:**
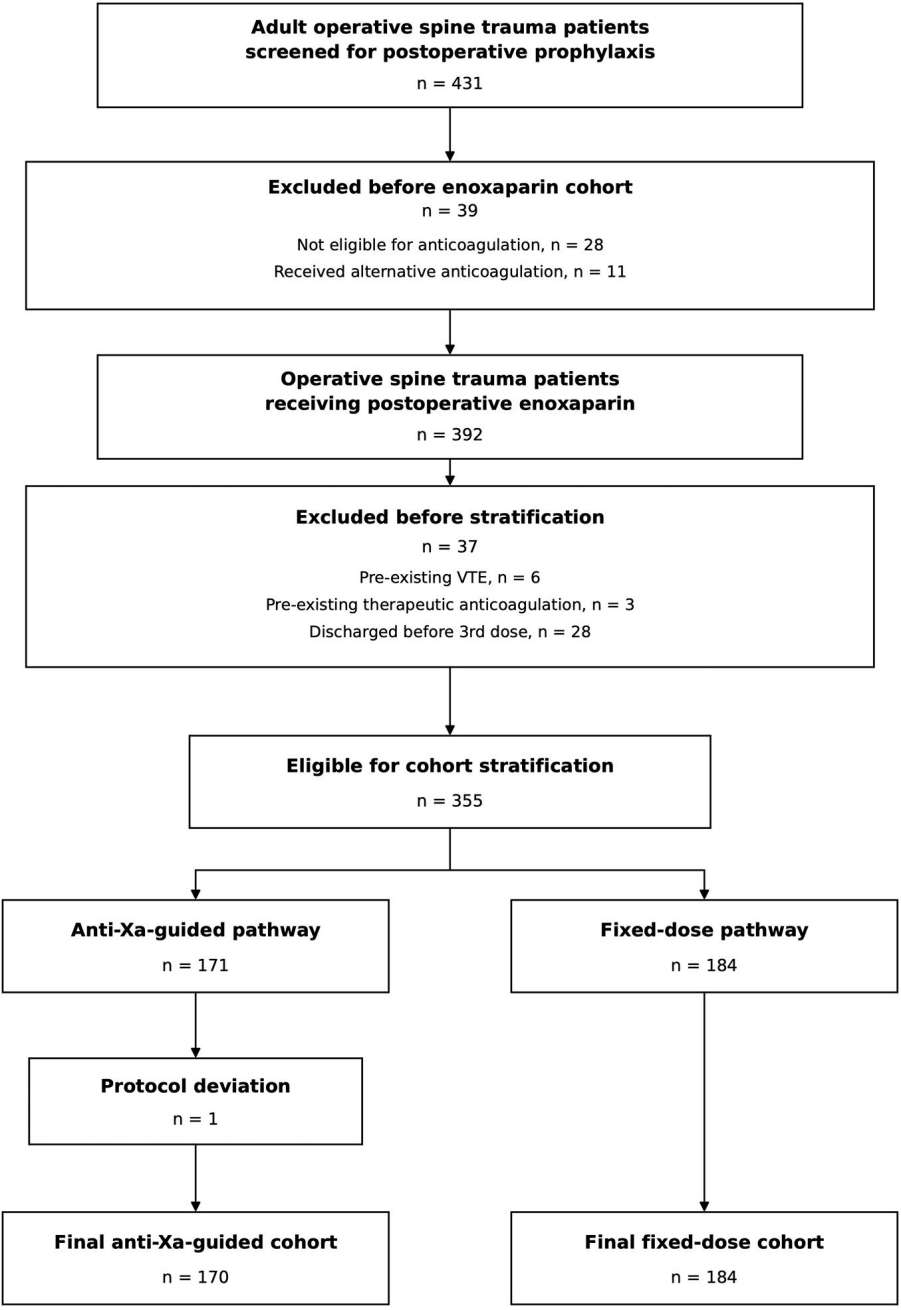
Patient selection flow diagram. Adult operative spine trauma patients were screened for postoperative pharmacologic prophylaxis. Patients not eligible for anticoagulation or receiving an alternative anticoagulation strategy were excluded before defining the postoperative enoxaparin cohort. Patients receiving postoperative enoxaparin were then excluded before cohort stratification if they had pre-existing VTE, pre-existing therapeutic anticoagulation, or discharge before the third postoperative enoxaparin dose. Eligible patients were stratified according to the postoperative enoxaparin prophylaxis pathway used during hospitalization. VTE, venous thromboembolism.

Between July 2022 and July 2024, 431 adult operative spine trauma patients were screened for postoperative pharmacologic prophylaxis. Thirty-nine patients were excluded before defining the postoperative enoxaparin cohort, including 28 patients who were not eligible for anticoagulation and 11 patients who received an alternative anticoagulation strategy. The remaining 392 patients received postoperative enoxaparin prophylaxis. Thirty-seven patients were excluded before cohort stratification, including 6 patients with pre-existing VTE, 3 patients receiving pre-existing therapeutic anticoagulation, and 28 patients discharged before completing the third postoperative enoxaparin dose. The remaining 355 patients were eligible for cohort stratification, including 171 patients in the anti-Xa-guided pathway and 184 patients in the fixed-dose pathway. One patient in the anti-Xa-guided pathway was excluded because a protocol timing deviation prevented valid anti-Xa classification. The final analytic cohort included 354 patients, with 170 in the anti-Xa-guided cohort and 184 in the fixed-dose cohort (Figure 1).

Demographic similarities were found between the two cohorts in regard to sex, age, and body habitus (Table 1). Both cohorts consisted of 65% male patients, with a median age of 55, and a median body weight of approximately 80 kg. Comorbidities in both groups, including diabetes, obesity, and smoking status, were similar between the two cohorts. Rates of prior thromboembolic events, clotting disorders, and use of anticoagulation or antiplatelet medications at admission were comparably low. The mechanisms of injury were also evenly distributed, with motor vehicle collisions and falls accounting for the majority of causes in both cohorts. The presence of spinal cord injury was more prevalent in the anti-Xa group than in the fixed-dose group (37% vs. 29%, *p* = 0.27) (Table 1). Additionally, patients in the fixed-dose cohort had higher Glasgow Coma Scale scores at the time of admission (mean 14.6 vs. 14.1, *p* = 0.03) (Table 1).

**Table 1:** Characteristics of traumatic spine surgery cohorts by thromboembolism prophylaxis protocol.

Variables	Anti-Xa (*N* = 170)	Fixed Dose (*N* = 184)	*P* value
Age			0.83
Mean (SD)	51.2 (19.4)	51.6 (19.8)	
Median (Q1, Q3)	55.0 (34.0, 66.8)	55.0 (34.0, 67.0)	
Male	111 (65%)	119 (65%)	0.91
Weight (kg)			0.66
Mean (SD)	83.4 (21.1)	83.1 (21.7)	
Median (Q1, Q3)	80.5 (68.2, 94.2)	80.0 (68.0, 91.8)	
BMI			0.55
Mean (SD)	28.3 (6.6)	27.9 (6.3)	
Median (Q1, Q3)	26.8 (24.4, 30.5)	26.5 (23.8, 31.0)	
**Diabetes**	36 (21%)	35 (19%)	0.69
**Obesity**	48 (28%)	57 (31%)	0.64
**Smoker**	27 (16%)	38 (21%)	0.27
**Clotting disorder**	2 (1%)	2 (1%)	1.00
**Prior DVT/PE**	5 (3%)	3 (2%)	0.49
**AC/AP**	23 (14%)	25 (14%)	1.00
**Admission Cr (ml/min)**			0.17
Mean (SD)	74.7 (33.9)	80.2 (35.4)	
Median (Q1, Q3)	72.9 (52.8, 96.4)	76.6 (54.7, 101.2)	
**Presence of pre-op neurologic deficit affecting ambulation**	74 (44%)	70 (38%)	0.33
Mechanism			0.97
Autoped	11 (6%)	11 (6%)	
Fall	73 (43%)	79 (43%)	
GSW	3 (2%)	3 (2%)	
MCC	3 (2%)	4 (2%)	
MVC	75 (44%)	78 (42%)	
other	5 (3%)	9 (5%)	
GCS			**0**.**03**
Mean (SD)	14.1 (2.1)	14.6 (1.3)	
Median (Q1, Q3)	15.0 (15.0, 15.0)	15.0 (15.0, 15.0)	
Spinal Cord Injury			0.27
ASIA A	27 (16%)	16 (9%)	
ASIA B	4 (2%)	3 (2%)	
ASIA C	11 (6%)	10 (5%)	
ASIA D	21 (12%)	24 (13%)	
No	107 (63%)	131 (71%)	
Other Injuries	57 (34%)	48 (26%)	0.13
TBI	19 (11%)	21 (11%)	1.00
Pelvic fracture	16 (9%)	14 (8%)	0.57
UE long bone fracture	11 (6%)	8 (4%)	0.48
LE long bone fracture	20 (12%)	16 (9%)	0.38
Solid organ injury	17 (10%)	16 (9%)	0.72

Bolded *p* values indicate *p* < 0.05 and are provided descriptively.

SD, standard deviation, Q1, first quartile, Q3, third quartile, TBI, traumatic brain Injury, GCS, Glasgow Coma Scale.

### Operative intervention and perioperative course

The time to surgical intervention, lumbar drain use, and blood loss were comparable between the two cohorts (Table 2). The mean number of fused levels was higher in the anti-Xa cohort (4.6 vs. 4.1, *p* = 0.04). Post-operative drain output was greater in the anti-Xa group (median 253 mL vs. 195 mL, *p* = 0.05).

**Table 2:** Summary of other variables and outcomes.

Variables	Anti-Xa (*N* = 170)	Fixed Dose (*N* = 184)	*P* value
Time to OR (days)			0.28
Mean (SD)	1.2 (1.6)	1.5 (2.2)	
Median (Q1, Q3)	1.0 (0.0, 1.0)	1.0 (0.0, 2.0)	
Blood loss (EBL)			0.08
Mean (SD)	404.1 (504.4)	333.7 (379.1)	
Median (Q1, Q3)	250.0 (150.0, 500.0)	200.0 (100.0, 437.5)	
Number of surgical levels			**0** **.** **04**
Mean (SD)	4.6 (2.2)	4.1 (2.0)	
Median (Q1, Q3)	4.0 (3.0, 6.0)	4.0 (3.0, 5.0)	
Approach			0.11
Anterior	18 (11%)	22 (12%)	
Posterior	131 (77%)	151 (82%)	
Both	21 (12%)	11 (6%)	
CTL recode w/o S			**0**.**02**
Cervical	66 (39%)	72 (39%)	
Cervical-thoracic	22 (13%)	14 (8%)	
Cervical-thoracic-lumbar	2 (1%)	0 (0%)	
Lumbar	6 (4%)	17 (9%)	
Thoracic	21 (12%)	36 (20%)	
Thoraco-lumbar	53 (31%)	45 (24%)	
**Laminectomy defect**	52 (31%)	49 (27%)	0.41
**Surgical drain output (mL)**			0.05
	350.8 (363.3)	296.8 (386.7)	
	252.5 (90.5, 498.8)	195.0 (28.8, 417.5)	
**Lumbar drain use**	22 (13%)	19 (10%)	0.51
**Post-op EDH requiring return to OR**	0 (0%)	0 (0%)	1.00
**Ambulatory post-op**	88 (52%)	118 (64%)	**0**.**02**
**Re-dose required**	47 (28%)	0	NA
Number of re-doses			NA
0	123 (73%)	0	
1	34 (20%)	0	
2	8 (5%)	0	
3	4 (2%)	0	
Post-op bleeding requiring transfusion (after starting enoxaparin)	25 (15%)	16 (9%)	0.10
Blood products transfused (mL)			0.56
Mean (SD)	712.1 (587.9)	800.4 (597.0)	
Median (Q1, Q3)	602.0 (300.0, 902.0)	551.0 (300.0, 1201.0)	
**Post-op VTE**	2 (1%)	3 (2%)	1.00
**Post-op DVT**	0 (0%)	2 (1%)	0.50
**Post-op PE**	2 (1%)	1 (1%)	0.61
LOS			**0**.**01**
Mean (SD)	12.0 (11.7)	9.6 (7.7)	
Median (Q1, Q3)	9.0 (5.2, 15.0)	7.0 (4.0, 12.0)	

Bolded *p* values indicate *p* < 0.05 and are provided descriptively.

SD, standard deviation; Q1, first quartile; Q3, third quartile; EDH, epidural hematoma; DVT, deep vein thrombosis; PE, pulmonary embolism; LOS, length of stay; POD, postoperative day; UE, upper extremity; LE lower extremity; EDH, epidural hematoma; NA, Not applicable.

### Anti-Xa monitoring and dose adjustment

Of the 170 patients managed with the anti-Xa protocol, 47 patients (28%) had anti-Xa activity below the prophylactic target range after initial weight-based dosing and underwent protocol-based dose escalation. Of those requiring redosing, 12 patients (25.5%) needed multiple dose escalations. Only four patients required three dose adjustments to reach the target anti-Xa range (Table 2). Following dose escalation, 4 of 47 patients (9%) subsequently required dose reduction for anti-Xa activity above the target range.

In exploratory multivariable analysis, higher body weight was associated with greater likelihood of requiring redosing (adjusted OR 1.05 per kg, 95% CI 1.00–1.10, *p* = 0.05). Notably, patients with traumatic brain injury (TBI) were also more likely to require redosing (adjusted OR 3.70, 95% CI 1.32–10.56, *p* = 0.01), while those with preoperative neurologic deficits were less likely to need adjustments (adjusted OR 0.06, 95% CI 0.003–0.38, *p* = 0.01) (Supplementary Table S1).

Within the anti-Xa-guided cohort, clinical outcomes were compared between patients who required dose escalation and those who did not (Table 3). Bleeding requiring transfusion occurred in 10 of 47 patients (21%) who required dose escalation and 15 of 123 patients (12%) who did not require dose escalation (*p* = 0.150). No postoperative epidural hematomas requiring return to the operating room occurred in either subgroup. Symptomatic VTE occurred in 0 of 47 patients requiring dose escalation and 2 of 123 patients (2%) without dose escalation (*p* = 1.000). No symptomatic DVT occurred in either subgroup, and symptomatic PE occurred in 0 of 47 patients requiring dose escalation and 2 of 123 patients (2%) without dose escalation (*p* = 1.000). Among patients who underwent dose escalation, 4 of 47 patients (9%) subsequently required dose reduction.

**Table 3:** Outcomes within the anti-Xa-guided cohort by dose escalation status.

Variables	Dose escalation (*N* = 47)	No dose escalation (*N* = 123)	*P* value
Bleeding requiring transfusion	10 (21%)	15 (12%)	0.150
Postoperative EDH requiring return to OR	0 (0%)	0 (0%)	NA
Symptomatic VTE	0 (0%)	2 (2%)	1.000
Symptomatic DVT	0 (0%)	0 (0%)	NA
Symptomatic PE	0 (0%)	2 (2%)	1.000
Dose reduction required after escalation	4 (9%)	NA	NA

EDH, epidural hematoma; VTE, venous thromboembolism; DVT, deep vein thrombosis; PE, pulmonary embolism; NA, not applicable.

*P* values were calculated using Fisher's exact test and are provided descriptively. NA was reported when no between-group comparison was applicable.

### VTE and bleeding outcomes

Symptomatic postoperative VTE was rare and similar between groups, occurring in 2 of 170 patients (1.1%) in the anti-Xa cohort and 3 of 184 patients (1.6%) in the fixed-dose cohort (*p* = 1.00). DVT occurred in 0 of 170 patients vs. 2 of 184 patients (1.1%; *p* = 0.50), and pulmonary embolism occurred in 2 of 170 patients (1.2%) vs. 1 of 184 patients (0.5%; *p* = 0.61). Because only five symptomatic postoperative VTE events occurred, regression modeling was not performed for VTE. VTE was analyzed descriptively using crude counts, percentages, and Fisher's exact test. No postoperative spinal epidural hematomas were observed in either group. In multivariable analysis, anti-Xa-guided dosing was not associated with bleeding requiring transfusion (Table 4).

**Table 4:** Univariable and multivariable analysis for the outcomes.

Categorical Outcomes	Anti-Xa (*n* = 170)	Fixed-Dose (*n* = 184)	OR [95% CI]	*P* value*	Adjusted OR [95% CI]	*P* value**
Post-op bleeding requiring transfusion (after starting enoxaparin)	25 (15%)	16 (9%)	1.81 [0.94, 3.58]	0.08	1.68 [0.84, 3.46]	0.15

Continuous Outcomes	Anti-Xa (*n* = 170)	Fixed-Dose (*n* = 184)	*β* [95% CI]	*P* value*	Adjusted *β* [95% CI]	*P* value**
Surgical drain output (mL)			54.04 [−24.55, 132.63]	0.18	26.50 [−49.07, 102.07]	0.49
Mean (SD)	351 (363)	297 (387)				
Median (Q1, Q3)	253 (91, 499)	195 (29, 418)				
Blood products transfused (mL)^a^			−88.32 [−471.31, 294.68]	0.64	−144.74 [−496.01, 206.54]	0.41
Mean (SD)	712 (588)	800 (597)				
Median (Q1, Q3)	602 (300, 902)	551 (300, 1,201)				
LOS			2.41 [0.35, 4.46]	**0**.**02**	0.93 [−0.89, 2.74]	0.32
Mean (SD)	12.0 (11.7)	9.6 (7.7)				
Median (Q1, Q3)	9.0 (5.2, 15.0)	7.0 (4.0, 12.0)				

Bolded *p* values indicate *p* < 0.05 and are provided descriptively.

LOS, length of stay; SD, standard deviation; Q1, first quartile; Q3, third quartile; OR = odds ratio comparing the anti-Xa protocol vs. observation; *β*, regression coefficient in linear models, representing the mean difference in the outcome between the anti-Xa group and the observation group; CI, confidence interval.

* *P* values are derived from univariable regression models that only included the group variable.

** *P* values are derived from multivariable regression models that included the group variable and prespecified potential confounders, as described in the Statistical Analysis section.

a The values are reported for the 41 subjects (25 in the anti-Xa group and 16 in the observation group) who had transfusion.

### Functional outcomes and hospital course

After multivariable adjustment, hospital length of stay did not differ between groups (adjusted *β*, 0.93; 95% CI, −0.89 to 2.74; *p* = 0.32), and bleeding requiring transfusion also did not differ (adjusted OR, 1.68; 95% CI, 0.84–3.46; *p* = 0.15) (Table 4). In crude analysis, ambulation at discharge was higher in the fixed-dose group than in the anti-Xa cohort (64% vs. 52%; *p* = 0.02).

## Discussion

Patients with traumatic spinal injuries are at substantially increased risk for VTE, with postoperative rates of approximately 8 to 10% reported in prior series despite chemoprophylaxis, compared with much lower rates in typical elective degenerative spine surgery cohorts (3–6). In the present study, symptomatic postoperative VTE was rare in both groups, occurring in 1.1% of patients managed with anti-Xa monitoring and 1.6% of patients receiving standard fixed dosing. These low incidence rates likely reflect the effect of an aggressive, multimodal prophylaxis strategy that included weight-based enoxaparin initiated on postoperative day one, routine use of sequential compression devices, and early mobilization. Similar protocols have been associated with reduced thromboembolic events in spine surgery. Cox et al. (30) observed a reduction in VTE to approximately 1% after implementation of early multimodal prophylaxis that included intraoperative and same-day unfractionated heparin without increased epidural hematoma risk (30). Massaro et al. (16) reported a VTE incidence of 0.21% among more than 4,700 spine surgery patients managed with early chemoprophylaxis, sequential compression devices, and early ambulation (16). Taken together, these data support the clinical rationale for early, structured prophylaxis in high-risk spine trauma populations.

Prior trauma studies support the use of anti-Xa-guided or individualized enoxaparin dosing to identify patients below prophylactic target ranges and improve target attainment, although the relationship between target attainment and clinically apparent VTE remains uncertain. The prophylactic anti-Xa range used in the present study was selected based on established trauma monitoring targets. The Western Trauma Association guidelines state that consensus anti-Xa targets for trauma prophylaxis are 0.2 to 0.4 IU/mL for peak levels or 0.1 to 0.2 IU/mL for trough levels (17). This peak range has also been used in multiple trauma anti-Xa dose-adjustment protocols, including Costantini et al. (23), Singer et al. (31), Karcutskie et al. (24), and Bigos et al. (25) (23–25, 31). Therefore, we used 0.2 to 0.4 IU/mL as an established prophylactic peak anti-Xa monitoring range while continuing to interpret target attainment as a pharmacodynamic endpoint rather than definitive evidence of clinical VTE reduction. Constantini et al. (23) and Singer et al. (31) reported frequent below-target anti-Xa levels with standard enoxaparin dosing and the need for dose escalation in many trauma patients (23, 31). Bigos et al. (25) and Taylor et al. (26) reported high rates of target attainment with weight-based or individualized dosing strategies, but did not show clear reductions in clinically apparent VTE rates (25, 26). Karcutskie et al. (24) further highlighted this uncertainty, finding similar VTE rates between anti-Xa-adjusted and fixed-dose groups and no reduction in VTE among patients who achieved prophylactic anti-Xa levels (24). Our findings extend this literature to postoperative spine trauma patients, in whom anti-Xa monitoring identified a substantial subset below the prophylactic target range despite initial weight-based dosing, while symptomatic VTE rates remained low and similar between groups. Collectively, these data support anti-Xa monitoring as a strategy for identifying below-target prophylactic anti-Xa activity, while emphasizing that larger prospective studies are needed to determine whether improved target attainment translates into fewer clinically apparent VTE events. Future studies incorporating routine surveillance imaging could also clarify whether targeted prophylaxis affects asymptomatic VTE in postoperative spine trauma patients.

The proportion of patients requiring dose escalation in our anti-Xa cohort provides clinically relevant pharmacodynamic insight for neurosurgical practice. Nearly one third of monitored patients (28%) remained below the prophylactic anti-Xa target range after initial weight-based dosing. These patients required at least one increase in enoxaparin dose, and approximately one in five required multiple adjustments to achieve the target range. Although this redosing rate is lower than the 54% to 71% reported in trauma studies using uniform 30 mg twice-daily regimens (23, 32, 33), it highlights the substantial interpatient variability that persists even when weight is incorporated. Higher body weight was associated with a greater likelihood of requiring redosing. Notably, patients with traumatic brain injury had an approximately threefold higher likelihood of requiring dose adjustment, whereas those with preoperative neurological deficits related to their spinal injury were less likely to need escalation. These findings suggest that certain patient subgroups, particularly those with higher body weight or concomitant TBI, may be more likely to require protocol-based dose escalation after initial weight-based dosing. Further work to refine which patients are most likely to remain below the prophylactic anti-Xa target range may help define where targeted anti-Xa monitoring is most clinically useful.

Equally important, anti-Xa-guided dose adjustment was not associated with an observed increase in clinically measured bleeding outcomes in this high-risk surgical population. No postoperative spinal epidural hematomas occurred in either group, and bleeding requiring transfusion did not differ between anti-Xa–guided and fixed-dose patients after multivariable adjustment. Within the anti-Xa-guided cohort, patients requiring dose escalation also had no symptomatic VTE events, although bleeding requiring transfusion was numerically higher and should be interpreted cautiously given the small subgroup size. Crude analyses showed longer hospital stays and lower ambulation rates in the anti-Xa cohort; however, length of stay did not differ after multivariable adjustment. The unadjusted differences in hospital course likely reflect baseline clinical and operative differences between cohorts, as patients in the monitored group had slightly lower Glasgow Coma Scale scores at presentation and underwent fusion across a greater number of levels. Our results align with reports showing low observed rates of clinically significant bleeding after early chemoprophylaxis in spine and spinal cord injury populations. Dhillon et al. (15) observed no increase in epidural hematoma among nearly 7,000 spine surgery patients who did or did not receive chemoprophylaxis, with rates of 0.20% and 0.18%, respectively (15). Massaro et al. (16) reported a 0.10% epidural hematoma rate with early enoxaparin in a large spine surgery cohort (16). In traumatic spinal cord injury, Chang et al. (19) reported that initiating heparinoids within 48 h of admission was not associated with intraspinal hematoma expansion, even among surgically treated patients (19). Taken together, these studies and our findings suggest that structured prophylaxis regimens after spine trauma surgery can be implemented with low observed rates of clinically significant bleeding when paired with careful postoperative monitoring.

Some aspects of this study warrant consideration. The analysis was retrospective and conducted at a single academic Level I trauma center, similar to many of the foundational studies on chemoprophylaxis and anti-Xa monitoring in trauma and spine surgery (15, 16, 23, 24). As with those reports, this design may limit generalizability, but reflects how prophylaxis is implemented in contemporary neurosurgical practice. Although our cohort of 354 operative spine trauma patients is large for this population, the absolute number of symptomatic VTE and epidural hematoma events was low, which limits the precision of rare-event risk estimates. Because postoperative spinal epidural hematoma is rare, with reported rates around 0.1% to 0.2% even in large spine surgery cohorts, the absence of observed events in this cohort should be interpreted cautiously and cannot exclude a small increase in risk (15, 16). VTE events in our study were identified on the basis of clinical suspicion with confirmatory imaging, rather than routine surveillance, an approach used in many spine trauma and spine surgery studies that similarly report low symptomatic VTE rates (3, 5, 16). This method likely underestimates asymptomatic thrombosis but aligns with real-world neurosurgical care and emphasizes clinically meaningful outcomes. Because multiple secondary and exploratory analyses were performed, findings beyond the primary symptomatic VTE comparison should be interpreted as hypothesis-generating. Finally, although multivariable models adjusted for prespecified clinical covariates, unmeasured confounding inherent to complex trauma care may remain. Therefore, the observed associations should not be interpreted as evidence that anti-Xa-guided dose adjustment causally reduced VTE.

Despite these design considerations, the present study provides clinically relevant data for spine surgeons considering individualized prophylaxis after traumatic spine surgery. Weight-based enoxaparin initiated on postoperative day one, combined with mechanical prophylaxis and early mobilization, was associated with low symptomatic VTE rates in this cohort. Anti-Xa monitoring also identified a meaningful subset of patients who remained below the prophylactic target range after initial weight-based dosing and required protocol-based dose escalation, particularly among patients with higher body weight or concomitant TBI. These findings support the feasibility of incorporating anti-Xa-guided enoxaparin titration into multimodal prophylaxis strategies for postoperative spine trauma patients, as the protocol identified patients below the prophylactic target range and allowed dose adjustment without an observed increase in bleeding requiring transfusion. Future multicenter prospective studies should determine whether improved anti-Xa target attainment reduces clinical VTE events and identify which patients are most likely to require monitoring or protocol-based adjustment. Such work should also better define the balance between target attainment and rare but clinically important bleeding risks.

## Data Availability

The raw data supporting the conclusions of this article will be made available by the authors, without undue reservation.
