# Supplementary material for: Weight-based, anti-Xa guided enoxaparin after spine trauma surgery: target attainment and safety in a retrospective cohort
Source: Front Surg. 2026 Jun 24;13:1764591. doi: 10.3389/fsurg.2026.1764591 (PMC13341635; doi:10.3389/fsurg.2026.1764591)
Supplement: Supplementary file 1 [file Table1.docx]

**Supplementary Table S1. Univariable and multivariable analysis for identifying risk factors for requiring redose on the anti-Xa group.**

| **Factors** | **Redose required** | | **P value^*^** | **Adjusted OR**  **[95% CI]^†^** | **P value^**^** |
| --- | --- | --- | --- | --- | --- |
|  | **Yes (N=47)** | **No (N=123)** |  |  |  |
| **Age** |  |  | 0.746 | 1.01  [0.99, 1.03] | 0.41 |
| Mean (SD) | 52.2 (19.2) | 50.8 (19.5) |  |  |  |
| Median (Q1, Q3) | 56.0 (38.0, 66.5) | 55.0 (34.0, 66.5) |  |  |  |
| **Male** | 35 (74%) | 76 (62%) | 0.15 | 1.18  [0.42, 3.33] | 0.76 |
| **Weight (kg)** |  |  | 0.019 | 1.05  [1.00, 1.10] | 0.05 |
| Mean (SD) | 90.4 (26.3) | 80.7 (18.2) |  |  |  |
| Median (Q1, Q3) | 85.0 (77.1, 99.3) | 76.8 (67.9, 91.5) |  |  |  |
| **BMI** |  |  | 0.121 | 0.90  [0.76, 1.05] | 0.19 |
| Mean (SD) | 29.6 (8.1) | 27.8 (5.9) |  |  |  |
| Median (Q1, Q3) | 27.7 (25.4, 31.9) | 26.7 (24.1, 30.1) |  |  |  |
| **Diabetes** | 8 (17%) | 28 (23%) | 0.53 | 0.65  [0.25, 1.56] | 0.35 |
| **Obesity** | 17 (36%) | 31 (25%) | 0.183 | 1.01 [0.31, 3.07] | 0.99 |
| **Smoker** | 5 (11%) | 22 (18%) | 0.349 | 0.70  [0.21, 1.95] | 0.52 |
| **Clotting disorder** | 1 (2%) | 1 (1%) | 0.478 | - | - |
| **-Prior DVT/PE** | 2 (4%) | 3 (2%) | 0.617 | 2.84  [0.35, 19.31] | 0.28 |
| **AC/AP** | 6 (13%) | 17 (14%) | 1 | 1.05  [0.34, 2.92] | 0.92 |
| **Admission Cr (ml/min)** |  |  | 0.449 | 1.01  [1.00, 1.02] | 0.25 |
| Mean (SD) | 78.0 (35.3) | 73.5 (33.4) |  |  |  |
| Median (Q1, Q3) | 77.9 (60.0, 94.8) | 72.3 (50.9, 97.2) |  |  |  |
| **Presence of pre-op neurologic deficit affecting ambulation** | 20 (43%) | 54 (44%) | 1 | 0.06  [0.003, 0.38] | **0.01** |
| **Mechanism** |  |  | 0.939 |  |  |
| Fall | 19 (40%) | 54 (44%) |  | referent level |  |
| MVC/MCC | 23 (49%) | 55 (45%) |  | 0.84  [0.37, 1.90] | 0.68 |
| Auto-ped, GSW, Other | 5 (11%) | 14 (11%) |  | 0.96  [0.26, 3.08] | 0.95 |
| **GCS** |  |  | 0.89 | 1.06  [0.90, 1.26] | 0.51 |
| Mean (SD) | 13.9 (2.3) | 14.1 (2.1) |  |  |  |
| Median (Q1, Q3) | 15.0 (15.0, 15.0) | 15.0 (15.0, 15.0) |  |  |  |
| **Spinal Cord Injury** | 21 (45%) | 42 (34%) | 0.218 | 1.24  [0.58, 2.61] | 0.58 |
| **Other Injuries** | 16 (35%) | 41 (33%) | 0.857 | 0.52  [0.17, 1.36] | 0.20 |
| **TBI** | 10 (21%) | 9 (7%) | 0.014 | 3.70  [1.32, 10.56] | **0.01** |
| **Pelvic fracture** | 3 (6%) | 13 (11%) | 0.561 | 0.52  [0.11, 1.85] | 0.35 |
| **UE long bone fracture** | 2 (4%) | 9 (7%) | 0.729 | 0.56  [0.08, 2.59] | 0.50 |
| **LE long bone fracture** | 7 (15%) | 13 (11%) | 0.428 | 1.38  [0.44, 3.99] | 0.57 |
| **Solid organ injury** | 5 (11%) | 12 (10%) | 1 | 1.00  [0.28, 3.18] | 0.99 |

Bolded p-value indicates statistical significance.

Abbreviations: SD = standard deviation; Q1 = first quartile; Q3 = third quartile; OR=odds ratio.

^*^ P values are from Kruskal-Wallis test for continuous variables and Fisher’s exact test for categorical variables.

^†^ OR stands for the odds ratio contrasting the categories for a categorical factor or associated with one unit increase for a continuous factor. Odds means the odds of requiring redose.

^**^ P values are from the multivariable regression model that includes each factor and all possible confounders with P<0.2 from the univariable test. Note that due to the high correlation between weight and BMI (correlation coefficient=0.89), only weight is included in the multivariable regression model to avoid numerical issue.

- Due to insufficient information, the factor `clotting disorder` cannot be included in the regression model.

**Supplementary Table S2. Univariable and multivariable analysis for identifying risk factors for the number of redoses required.**

| **Factors** | **Number of Redoses Required** | | | **P value^*^** | **Adjusted RR**  **[95% CI] ^†^** | **P value^**^** |
| --- | --- | --- | --- | --- | --- | --- |
|  | **0 (N=123)** | **1 (N=34)** | **2/3 (N=12)** |  |  |  |
| **Age** |  |  |  | 0.85 | 1.00 [0.99, 1.02] | 0.77 |
| Mean (SD) | 50.8 (19.5) | 52.9 (20.1) | 49.4 (17.3) |  |  |  |
| Median (Q1, Q3) | 55.0 (34.0, 66.5) | 56.5 (37.0, 68.0) | 49.5 (41.2, 62.2) |  |  |  |
| **Male** | 76 (62%) | 24 (71%) | 10 (83%) | 0.27 | 1.53 [0.82, 3.02] | 0.19 |
| **Weight (kg)** |  |  |  | **0.046** | 1.01 [1.00, 1.03] | **0.03** |
| Mean (SD) | 80.7 (18.2) | 89.6 (29.7) | 92.1 (15.7) |  |  |  |
| Median (Q1, Q3) | 76.8 (67.9, 91.5) | 84.5 (72.8, 95.3) | 86.5 (79.0, 105.7) |  |  |  |
| **BMI** |  |  |  | 0.17 | 1.02 [0.98, 1.06] | 0.28 |
| Mean (SD) | 27.8 (5.9) | 29.4 (9.3) | 29.9 (4.2) |  |  |  |
| Median (Q1, Q3) | 26.7 (24.1, 30.1) | 27.3 (24.5, 31.6) | 29.2 (26.6, 33.0) |  |  |  |
| **Diabetes** | 28 (23%) | 6 (18%) | 2 (17%) | 0.85 | 0.70 [0.31, 1.42] | 0.34 |
| **Obesity** | 31 (25%) | 11 (32%) | 5 (42%) | 0.37 | 0.85 [0.37, 1.91] | 0.68 |
| **Smoker** | 22 (18%) | 3 (9%) | 2 (17%) | 0.47 | 0.90 [0.37, 1.98] | 0.8 |
| **Clotting disorder** | 1 (1%) | 0 (0%) | 0 (0%) | 1.00 | - | - |
| **Prior DVT/PE** | 3 (2%) | 1 (3%) | 0 (0%) | 1.00 | 0.95 [0.05, 5.86] | 0.96 |
| **AC/AP** | 17 (14%) | 4 (12%) | 1 (8%) | 1.00 | 0.80 [0.29, 1.90] | 0.63 |
| **Admission Cr (ml/min)** |  |  |  | 0.64 | 1.01 [1.00, 1.01] | 0.19 |
| Mean (SD) | 73.5 (33.4) | 75.5 (35.5) | 84.2 (36.8) |  |  |  |
| Median (Q1, Q3) | 72.3 (50.9, 97.2) | 73.7 (63.1, 88.3) | 90.3 (53.2, 104.0) |  |  |  |
| **Presence of pre-op neurologic deficit affecting ambulation** | 54 (44%) | 15 (44%) | 5 (42%) | 1.00 | 0.83 [0.46, 1.48] | 0.53 |
| **Mechanism** |  |  |  | 0.61 |  |  |
| Fall | 54 (44%) | 14 (41%) | 4 (33%) |  | referent level |  |
| MVC/MCC | 55 (45%) | 15 (44%) | 8 (67%) |  | 1.13 [0.61, 2.10] | 0.7 |
| Auto-ped, GSW, Other | 14 (11%) | 5 (15%) | 0 (0%) |  | 0.79 [0.25, 2.15] | 0.67 |
| **GCS** |  |  |  | 0.31 | 1.02 [0.90, 1.17] | 0.77 |
| Mean (SD) | 14.1 (2.1) | 13.6 (2.6) | 14.7 (1.2) |  |  |  |
| Median (Q1, Q3) | 15.0 (15.0, 15.0) | 15.0 (14.0, 15.0) | 15.0 (15.0, 15.0) |  |  |  |
| **Spinal Cord Injury** | 42 (34%) | 16 (47%) | 5 (42%) | 0.41 | 1.21 [0.68, 2.14] | 0.5 |
| **Other Injuries** | 41 (33%) | 13 (39%) | 3 (25%) | 0.64 | 0.67 [0.29, 1.41] | 0.31 |
| **TBI** | 9 (7%) | 9 (26%) | 1 (8%) | **0.01** | 1.77 [0.81, 3.70] | 0.13 |
| **Pelvic fracture** | 13 (11%) | 2 (6%) | 1 (8%) | 0.81 | 0.84 [0.27, 2.14] | 0.73 |
| **UE long bone fracture** | 9 (7%) | 1 (3%) | 1 (8%) | 0.65 | 0.76 [0.17, 2.41] | 0.67 |
| **LE long bone fracture** | 13 (11%) | 4 (12%) | 3 (25%) | 0.31 | 1.64 [0.74, 3.44] | 0.19 |
| **Solid organ injury** | 12 (10%) | 4 (12%) | 1 (8%) | 0.90 | 1.13 [0.43, 2.65] | 0.79 |

Bolded p-value indicates statistical significance.

Abbreviations: SD = standard deviation; Q1 = first quartile; Q3 = third quartile; RR=Rate ratio.

^*^ P values are from Kruskal-Wallis test for continuous variables and Fisher’s exact test for categorical variables.

^†^ RR stands for the rate ratio contrasting the categories for a categorical factor or associated with one unit increase for a continuous factor.

^**^ P values are from the multivariable negative binomial regression model that includes each factor and all possible confounders with P<0.2 from the univariable test. Note that due to the high correlation between weight and BMI (correlation coefficient=0.89), only weight is included in the multivariable regression model to avoid numerical issue.

- Due to insufficient information, the factor `clotting disorder` cannot be included in the regression model.

^∂^ One subject is excluded from the analysis due to missing number of redoses.
